# Ethical Decision Making in Autonomous Vehicles: The AV Ethics Project

**DOI:** 10.1007/s11948-020-00272-8

**Published:** 2020-10-13

**Authors:** Katherine Evans, Nelson de Moura, Stéphane Chauvier, Raja Chatila, Ebru Dogan

**Affiliations:** 1 Institut VEDECOM, 21 bis Allée des Marroniers, 78000 Versailles, France; 2 Sciences, Normes, Démocratie, Sorbonne Université, 1 Rue Victor Cousin, 75005 Paris, France; 3 ISIR, Sorbonne Université, 4 Place Jussieu, 75005 Paris, France

**Keywords:** Autonomous vehicles, Ethics, Decision making, Moral reasoning, Machine ethics

## Abstract

The ethics of autonomous vehicles (AV) has received a great amount of attention in recent years, specifically in regard to their decisional policies in accident situations in which human harm is a likely consequence. Starting from the assumption that human harm is unavoidable, many authors have developed differing accounts of what morality requires in these situations. In this article, a strategy for AV decision-making is proposed, the Ethical Valence Theory, which paints AV decision-making as a type of claim mitigation: different road users hold different moral claims on the vehicle’s behavior, and the vehicle must mitigate these claims as it makes decisions about its environment. Using the context of autonomous vehicles, the harm produced by an action and the uncertainties connected to it are quantified and accounted for through deliberation, resulting in an ethical implementation coherent with reality. The goal of this approach is not to define how moral theory requires vehicles to behave, but rather to provide a computational approach that is flexible enough to accommodate a number of ‘moral positions’ concerning what morality demands and what road users may expect, offering an evaluation tool for the social acceptability of an autonomous vehicle’s ethical decision making.

## Introduction

Autonomous vehicles (AVs) are shifting from prospect to imminent reality in the eyes of Original Equipment Manufacturers, government institutions, and the general public alike. As recent and somewhat grizzly events have revealed, this shift is not without risk, even as technology improves, accidents will continue to occur. The ethics of autonomous vehicles have thus quickly become a polemic subject, specifically in regards to the apparent pluralism of moral preference across a given society, and the so-called “social dilemma” of selecting a general decisionary maxim, even one as inoffensive as “minimise casualties” (Bonnefon et al. 2016). So, while the risks, vulnerabilities and dilemmas of the move towards autonomous driving have been identified, a practical, implementable solution must still be found. What steps are necessary to ensure the societal benefits promised by the advent of autonomous vehicles?

An important part of the solution undoubtedly lies in the decision-making of the autonomous vehicle itself. A major assumption and popular talking-point in the autonomous vehicle debate is the AV’s eventual capacity to eliminate human error from the traffic environment: no more drunk-driving, texting, sleeping, or otherwise preoccupied drivers on the road. Stronger still, in unavoidable crash scenarios, the autonomous vehicle is purported to make a deliberative decision as to how it will crash, supplanting the ineffective and irrational reactions of human drivers (Lin et al. 2017). This is a tall order to fill for any artificial decision process, let alone one that is acting within an environment as complex, volatile and unpredictable as any modern traffic community. In spite of these challenges, an implementable solution to effective and acceptable decision making in autonomous vehicles must be found. This article discusses one such strategy for AV decision-making, called the Ethical Valence Theory (EVT). The theory paints AV decision-making as a type of claim mitigation: different road users hold different moral claims on the vehicle’s behavior, and the vehicle must mitigate these claims as it makes decisions about its environment^1^. Specifically, it must find an optimal response to these claims in cases of unavoidable collision, or in ‘dilemma scenarios’; one which captures most efficiently the moral claims and relations which exist within the vehicle’s decision context, and aligns best with user expectations. The article will first address the larger context of autonomous vehicle ethics, then it will provide a conceptual introduction to the Ethical Valence Theory, and finally delve into the mathematical foundations for the application of the EVT in level 4 autonomous vehicles.

## Autonomous Vehicle Ethics: Risk Mitigation

The human traffic environment is one of volatility, uncertainty, cultural relativity, and in unfortunate cases, one of lethality. In effect, the number of deaths on the world’s roads remains unacceptably high, with an estimated 1.35 million people dying each year and up to 50 million injuries (World Health Organization 2018). In response, many stake-holders, institutions, and drivers have heralded autonomous vehicles (AVs) as the latest—if not the ultimate—advance towards accident-free roads. Yet, even the most optimistic long-term estimates of the impact of autonomous vehicles predict a 90% reduction in traffic-related accidents (Fagnant and Kockelman 2015; Airbib and Seba 2017; Gao et al. 2016). While this figure is unrivaled and impressive, the fact remains that lethal, serious and near-accidents will continue to occur, albeit less frequently, once autonomous vehicles are on the world’s roads, a fortiori in their early implementation stages when mixed-fleet traffic forces autonomous vehicles to interact with human drivers. In light of these predictions, it seems negligent to conceive of autonomous vehicles as purely innocuous road users. Physical, if not lethal harm will continue to be a feature of the traffic environment as AV implementation advances.

Consequently, the continued presence of harm in mixed fleet traffic environments has provoked two related responses in the literature. First, the harkening of these dilemma-type decision situations to ‘the trolley problem’ (Foot 1967), and second, a wealth of dilemma scenario analysis as to the types of decisional or ethics policies that would encompass how morality requires the AV to act in these sorts of dilemmas. Ostensibly, the question of the ideal ethics policy is an open and thorny one, bringing many meta-ethical and multidisciplinary considerations into play. For it seems that any robust decision as to the moral content that grounds an ethics policy is tacitly supported by a number of meta-considerations, such as the choice of the source of moral content, whether it be public and participative (Bonnefon et al. 2016; Greene et al. 2016), or from traditional western ethical paradigms such as utilitarianism (Lin et al. 2017), Rawlsian theories of justice (Leben 2017), or the doctrine of double effect (De Sio 2017; Keeling 2018). These discussions tend to be exacerbated by the apparent lack of ground-truth ethical principles across diverse societies (Noothigattu et al. 2018), and the failure of user expectations to closely align with any pre-existing moral theory (Awad et al. 2018). Additionally, there are questions pertaining to the relative roles of ethics and the law in deciding vehicle behavior. Particularly, whether the role of ethics can be subsumed by more careful legal analysis (Casey 2016), or whether the demands of legal responsibility will not simply resolve the question of the ideal ethics policy altogether, by requiring some degree of direct user participation, or mandatory ethics settings (Contissa et al. 2017; Millar et al. 2017; Danaher 2016). Finally, there are fundamental critiques of the use of the trolley problem as a cogent policy tool in the case of AVs (Himmelreich 2018; Keeling 2019; Nyholm and Smids 2016), and whether these individual decision-cases cannot be managed through the larger ethical analysis of autonomous vehicles as a disruptive technology (Epting 2018). Thus, in just a few short years, expert debate has spun an exceedingly immobilizing web around vehicles whose wheels are already hitting public roads, and the question of the ideal ethics policy remains glaringly open.

Yet, from this environment of moral uncertainty (Bhargava and Kim 2017) some elements of an ideal ethics policy appear. For instance, if we accept the claim that the wide-spread use and adoption of AVs is a necessary condition of the many societal benefits these vehicles are purported to provide (Bonnefon et al. 2016), then it is quite clear that any reasonable ethics policy of an autonomous vehicle cannot turn a blind eye to public acceptability. It would seem that for autonomous vehicles to truly become a morally optimal mode of transportation, their behaviour must track the various expectations of the users with which they interact, and the larger societies in which they are implemented. In one trivial sense, this constraint amounts to ensuring the user’s satisfaction and safety, and likely other prominent design values such as trust, accountability and transparency (Initiative 2016). As it concerns the vehicle’s ethics policy however, this claim would seem to provide a strong reason to prefer those moral theories which do not revise upon what is conventionally called ‘common sense morality’; or more weakly, a reason to dismiss any account of the moral good which fails to adequately capture generally held moral attitudes. If this is valid, then many popular accounts of morality, including most forms of deontological ethics, utilitarianism, and Rawlsian contractarianism, all appear to be in trouble; and if they survive, few will have retained the purity of their original shape, motivations, and scope. From this perspective, within the context of AV ethics, moral theory then seems at the very least beholden to, if not limited by, prevailing moral attitudes.

On the other hand, however, it seems just as morally problematic to disregard moral theory altogether, taking exclusive inspiration from the (moral) *‘wisdom of the crowd’* (Dignum 2019). Much support for this latter claim is drawn from a concern for the algorithmic implications of human moral failure: whether it be as a result of bias, prejudice, ignorance, irrationality, akrasia, or straightforward egoism, the behavior of human beings provides (training) data that is at the very least morally sub-optimal, and may border on morally unacceptable (Dignum 2019; Leben 2018). As some see it, artificial agents not only ought to avoid emulating these types of behaviors across their interactions, but may in themselves represent an opportunity for human moral improvement if they are instead designed to act as pure moral reasoners (Gips 1994; Arkin 2009; Grau 2006; Dietrich 2001). In this instance then, ethics policies based in pure accounts of moral theory may be morally permissible or perhaps required, despite their misalignment with user expectations, and despite their bringing what Derek Leben has called *‘morally superior robot villains’* into our everyday lives (Leben 2018). Then, from this vantage point, it would appear that public acceptability is something to be disregarded, if not improved upon by robotic technologies like autonomous vehicles.

Thus, an ideal ethics policy must, to some degree, resolve the inherent tension between these two factions, by striking a balance between public acceptability and moral requirement. It would seem that it must be just acceptable enough to garner trust and adoption from human users, but just moral enough to avoid echoing the most reprehensible of human inclinations. Likewise, an ideal ethics policy is not best defined as a computational answer to the trolley problem, but rather as a set of procedures that ought to be performed in sacrificial, or dilemma situations. Often, these situations exist in places where the law is silent, or in any case cannot currently provide a complete answer to whom the vehicle should privilege or sacrifice through its actions. The ethics of autonomous vehicles then sits squarely within these different lacunae, and regardless of the ethics policy implemented, it is important that the surrounding decisional architecture reflect and support this complexity, and not skirt or deny its depth. In this spirit, the following section will attempt to provide an architecture flexible enough to accommodate an array of these types of ethics policies, while leaving the question of the ‘right’ ethics policy open.

## The Philosophical Foundations of the Ethical Valence Theory

### Claims and Foundations

The philosophical approach behind the Ethical Valence Theory is best understood as a form of moral claim mitigation. The fundamental assumption is that any and every road user in the vehicle’s environment holds a certain claim on the vehicle’s behavior, as a condition of their existence in the decision context. In other words, every individual - from pedestrian to passenger - has a certain expectation as to how the vehicle will treat him or her in its deliberation, which underpinned by facts about individual welfare, provides a reason for the vehicle to behave in a certain way. Conceptually, the EVT paints autonomous vehicles as a form of ecological creature (Gibson and Crooks 1938; Gibson 1979), whose agency is directly influenced by the claims of its environment. Claims can vary in strength, for instance, a pedestrians claim to safety may be stronger than a passenger’s claim if the former is liable to be more seriously injured as a result of an impact with the AV. The goal of the autonomous vehicle is to (maximally) satisfy as many claims as possible as it moves through its environment, responding in proportion to the strength of each claim.

Analytically, individual claims can be understood as contributory or pro tanto reasons for the vehicle’s acting a certain way (Dancy 2004; Prichard 2002). Each claim acts as a contributory ‘ought’, meaning that the strength of a claim is directly relative to how strongly it ‘ought’ to respond to the individual’s claim, or his ‘moral pull’ (Nozick 1981). To take an example, in regular driving conditions, an autonomous vehicle has a reason, all things considered, to privilege the claim to safety of its passenger in its tactical decision-making. However, when a dilemma situation arises and an unavoidable collision is imminent, the vehicle will be faced with other reasons, all things considered, to privilege the claims to safety of other road users, such as pedestrians or cyclists. Since these reasons are in conflict, the vehicle must then decide which of these reasons is the strongest, and act on the strongest reason. Then, by responding to the strongest claim in its environment, the vehicle is doing what it ‘most ought’ to do, morally speaking.

Within the structure of the Ethical Valence Theory, the role of claim mitigation is to capture the contribution that normative ethics could make to autonomous vehicle decision-making. Claims, in other words, allow the vehicle to ascertain what morality requires in critical scenarios, by tracking how fluctuations in the welfare of road users affect the rightness or wrongness of an AV’s action. In many respects, this approach takes inspiration from the ‘competing claims’ model popular in distributive ethics (Nagel 2012; Voorhoeve 2014), and taken this way, we would initially have reason to view the Ethical Valence Theory as foundationally utilitarian, however this vision is incomplete. In effect, since the principal aim of the EVT is to provide an account of AV ethical decision-making which is sensitive to public acceptability, we must take seriously the idea that other potentially normatively relevant factors, such as agent-relative constraints and options (Kagan 1992) must feature in the theory’s foundational structure. We must also attempt this without falling into the appealing trap of supposing that this requires AVs to hold some particular moral status, or to possess popular prerequisites for personhood such as intentionality, subjectivity, or free will (Talbot et al. 2017; Bryson 2018; Wilks 2010)

In this spirit, it is plausible that if agent-relative constraints and options are normatively relevant at all in the context of autonomous vehicles, they are so if they track the expectations certain road users might have as to partiality on the part of the AV. In this sense, the passenger likely expects her AV to be partial to her interest and those of her family, even more so in situations where she is liable to be gravely injured or killed (Keeling et al. 2019).

This expectation may in part flow from a perception of the AV as a proxy or surrogate of her practical agency, acting on behalf of her in the traffic environment (Keeling et al. 2019; Johnson and Powers 2008; Millar et al. 2017). Thus, her autonomous vehicle may indeed exhibit the behavior of an unacceptable, *‘morally superior robot villain’* if it categorically fails to insulate her and her loved ones from harm, thereby neglecting the importance these special ties have for her. Importantly, the inclusion of a form of morally admirable partiality on the part of the AV towards its passenger need not, as is often implied in popular media, lead to a form of passenger-centric *exclusivism*, wherein the passenger’s interest constitutes the only normatively relevant factor which decides the rightness of the AV’s actions. Instead, it is enough to say that the fact that a certain human is the AV’s passenger constitutes one normatively relevant factor which must be addressed alongside facts about the individual welfare of all road users.

This bizarre mix at the factoral level of the EVT moves us away from utilitarianism and towards contractarian forms of foundational theory—and perhaps specifically Scanlonian contractualism—since these types of theory typically view the correct list of normatively relevant factors as those which would be agreed upon, consented to, or reasonably unobjectionable for suitably disposed and informed individuals acting in society (Kagan 1992; Scanlon 1998). Nevertheless, the choice of adopting (Scanlonian) contractualism as the EVT’s foundational theory has its limits. Importantly, the concept of claim mitigation as we have introduced it here, revolves around the idea that the AV’s role in ethical decision-making is to directly appraise the claims and interests of the individual road users in its environment. This does not coincide with the contractualist account of moral deliberation, wherein the agent typically appraises the reasons individuals have to reject the agent’s current motivational principles, thereby selecting that principle which provides reasons for action that no individual could reasonably reject. To use Shelley Kagan’s vocabulary, rules and principles, rather than the acts they promote, make up the ‘focal point’ of contractualist theories, and thus the perspective from which moral deliberation occurs (Kagan 1992). This deliberative step is missing in the actual decision procedure of the Ethical Valence Theory, and is instead accomplished by the human decision-makers involved the design process. In this sense, we can view this difference as a departure from Scanlon, requiring a division of cognitive labour between the designer and the machine which is clearly missing in Scanlon’s original theory. Or, complimentarily, we can view this deliberative step as the principal task of what is often called the ‘conceptual phase’ of intelligent artefact design, following the Value Sensitive Design methodology (Friedman et al. 2008). The EVT, once implemented into an autonomous vehicle, does not deliberate ‘across’ principles in order to find the most acceptable (or least rejectable) option, it simply acts on that principle which its human designers have chosen to implement, which may or may not satisfy the expressly Scanlonian conditions of reasonable rejection.

Instead, the main points of resemblance between contractualism and the foundational structure of Ethical Valence Theory are threefold: first, like contractualism, the EVT does not seek to revise upon common sense morality by providing a monistic account of what grounds moral value. It is instead a pluralist account of morality, leaving room for both the goodness of results, and special and general obligations in its account of normatively relevant factors. Second, like (Scanlonian) contractualism, the EVT abides by an impersonalist restriction which holds that “...in rejecting some moral principle, we cannot appeal to claims about the impersonal goodness or badness of outcomes” (Parfit 2011; Scanlon 1998). Finally, both contractualism and the Ethical Valence Theory abide by a further restriction of an ‘individualist’ nature, which holds that all moral reasons for action “...must appeal to the principle’s implications only for ourselves and for other single people...” (Parfit 2011; Scanlon 1998). These restrictions are valuable to the EVT in so far as they prevent the aggregation of claims in the vehicle’s ethical deliberation, thereby ensuring that the vehicle considers only direct changes in individual welfare, or each individual’s degree of claim satisfaction. What we are left with then resembles a pluralist form of act consequentialism which abides by contractualist constraints and principles.

### The Concept of ‘Valence’

If, from the perspective of normative ethics, the contractualist motivations of the Ethical Valence Theory serve principally to track common sense morality as it pertains to the case of autonomous vehicles, the notion of a ‘valence’, and its role within the theory, serves mainly to track (empirical) accounts of public acceptability. From the previous section, we have established that each road user holds a claim not to be harmed by the autonomous vehicle in the event of a collision, the strength of which varies in relation to the severity of an eventual injury incurred as a result of this collision. Critically, each road user also holds a specific valence, which varies in strength in relation to how that individual user’s identity corresponds to a number of set criteria^2^. Beyond the technical limitations of identification, data collection and processing, there are no specific criteria that ought to inform a valence, but can cover features like different age groups, socio-economic levels or professions following the line of the Moral Machines Experiment (Bonnefon et al. 2016), or cover forms of morally admirable partiality that might exist between the AV and its passenger(s) (Keeling 2019). Arguably, most of the criteria chosen to inform valences will remain polemic, and given space limitations, it would be unwise to attempt to rehearse the full extent of the debate here. What we will say is that any attempt to identify the moral limits of valence features, while certainly falling under the purview of moral philosophy, must also engage with the burgeoning world of so-called ethical design principles (Dignum 2019; Jobin et al. 2019), as well as emergent ethico-legal doctrines from various political institutions (Luetge 2017; On Ethics in Science and Technologies 2018), which taken together at least, may impose very strict limitations on what can count as admissible discriminatory features across road users. The German Parliament’s Ethics Commission on Automated and Connected Driving, for instance, explicitly forbids “...any distinction based on personal features (age, gender, physical or mental constitution)...” (Luetge 2017), which would seem to exclude all but a circumstantial categorization of individuals, discriminating only ‘cyclists’ from ‘pedestrians’, and other such ‘types’ of road user. To this end, categorization of road users across the lines of their relative ‘vulnerability’, as it is often accomplished in the field of traffic psychology, may succeed in satisfying such stringent conditions.

Faced with such exacting informational constraints then, both the appeal and the utility of valences may seem questionable. This sentiment may glean further support from two additional challenges posed by the selection of valence criteria. First, there is, from an acceptability standpoint at least, a clear relationship between decisional accuracy and robustness of information, which is simply to say that the more fine grained the valence criteria are, the more the resultant decisions of the AV will be able to track public acceptability (Kearns and Roth 2019). It would then seem that, ideally, valences should encompass all of the salient facts of an environment in order to afford an ideally informed decision. Ostensibly, these facts can be collected from a number of sources —for example, explicit input from the passenger, empirical studies, or data recovered from environmental perception, and vehicle-to-vehicle or vehicle-to-device communication—and can encompass a number of traits that are comparable across different road users: health status, age, income or occupation, among many other options. Beyond the already harrowing choice of deciding which features matter, there is the additional worry of failing to adequately distinguish *how* they matter. Taking the example of age, we can make the case that certain cultures may have values which place higher social importance on the elderly—for their wisdom, perspective, or other less instrumental reasons—while others seem to worship the cult of youth, and thus may be relatively more willing to sacrifice the elderly in the event of an AV collision. In both cases, preferential gerantophobia can be captured by local empirical research, and the valences will accordingly reflect such differences. Age, however, can just as easily feature in the robust appraisal of an individual’s welfare (and thus his ‘claim’), in so far as the elderly are liable to suffer higher instances of injury or death as a result of a collision (Liu et al. 2019). Thus in order to avoid ambiguity, it is important to reflect on the nature of the facts that form the basis of valences, both in the formulation of empirical studies and surveys, and in the analysis of the results.

A second hurdle that faces the notion of valence as we have described it is the unintended or collateral effects that the choice of certain features might have on the overall traffic environment. In his paper on autonomous vehicle ethics, Noah J. Goodall (2014) takes the example of an AV that is designed to discriminate between motorcyclists who are wearing helmets and motorcyclists who are not wearing helmets in the vehicle’s environment. In the case of an unavoidable accident, this can lead to an uncomfortable trade-off: in choosing to sacrifice the helmet-wearing motorcyclist, the vehicle minimizes harm by essentially targeting the least vulnerable road user. However, in doing so, it disincentives other motorcyclists from wearing helmets, thus indirectly generating greater risk in the overall traffic environment. Still, by choosing to sacrifice the motorcyclist without a helmet, it commits another type of error: by expressly valuing helmet-wearing motorcyclists, it unfairly targets unlawful road users, displaying an uncomfortable form of technological paternalism that may appear to ’punish’ those who do not follow the letter of the law. Here again, the interplay between an impartial claim to safety and a valence can add complexity to an AV’s decision. In light of the adverse effects that a ‘helmet/no-helmet’ criteria can have on the traffic environment, it might be wise to remove it from consideration, despite its having a clear effect on a road user’s claim to safety. However, by the same token, empirical research may be able to internalize this effect, by treating it as an issue of public acceptability, and not simply public safety. Unsurprisingly then, the design of valence criteria is highly contentious, precisely because it requires policy makers and engineers to identify a priori which relatively trivial facts might one day become the decisive factor in a life-or-death decision. However, designing a deliberation process that does not depend solely on valence-type considerations may help alleviate some of this pressure, specifically through the separation of moral claims from social acceptability at every level of the design process.

### Moral Profiles

The final conceptual piece of the Ethical Valence Theory is the notion of a ’moral profile’: a specific decision procedure or method which mitigates the different claims and valences of road users. Essentially, each moral profile provides a specific criterion of rightness: a maxim or rule which decides the rightness or wrongness of action options. In this way, a moral profile also dictates which claims the AV is sensitive to and when, and how those claims are affected by a given individual’s valence strength. While there are surely many ways to organize the mitigation process between valences and claims, one way to honour the special obligation the AV may have in regards to its passenger is to make a preliminary categorial separation between those users inside the AV and those outside of it, thereby painting claim mitigation as the balancing of the passenger(s) claim versus those in the AV’s surrounding environment. Then, there are a number of potential mitigations we could find across these two groups of interest: a risk-averse altruist moral profile would, for example, privilege that user who has the highest valence in the event of a collision, so long as the risk to the AV’s passenger is not severe. Intuitively, this type of profile supports the view that the passenger of the AV may be willing to incur some degree of harm in order to respond to the claims of other users in the traffic environment, but not so much that he will die or suffer seriously debilitating injuries as a result. A profile such as this might help dispel public concern about so-called ‘killer cars’, and bolster user confidence in AVs. Conversely, a threshold egoist type profile would privilege the AVs passenger, so long as the risk of harm to a user with a higher valence than the passenger is not severe. If the valence criteria were configured to address (and eventually prioritize) highly vulnerable road users, such as small children, the elderly or the disabled, a profile such as this might improve the societal acceptability of AVs.

In the end, there is no single profile that will once and for all resolve the moral and social dilemma of autonomous vehicles. Instead, the choice of moral profile, along with the choice of valence criteria, exist as different entry points for human control in an autonomous machine. Importantly, within this model, the public, too are given a role to play in the decisions an AV will make; but one that is mitigated by a responsiveness to moral urgency, and a fair and structured deliberation process on the part of the AV. It is this type of flexibility that we hope will ensure that the actions of an autonomous vehicle are acceptable, and not only moral.

Admittedly, the cogency of the Ethical Valence Theory is hampered by a familiar computational shortcoming: the reliance on the calculation of the degree of harm liable to be sustained by individual road users. In the literature on AV ethics, this is a recurring problem (Gerdes and Thornton 2015; Leben 2017; Lin et al. 2017), as many ethics policies which aim at harm or risk minimization lack the informational certainty necessary to effectively predict the harmfulness of individual collisions. In this respect, the Ethical Valence Theory is vulnerable to similar criticisms. In the absence of specific information related to elements such as the posture of occupants or the structural integrity of specific vehicles—compounded by uncertainty in the vehicle’s perception itself—estimating potential harm to road users will remain approximative and uncertain. However, despite this pitfall, the Ethical Valence Theory has the advantage of avoiding complete reliance on harm probability within ethical decision- making. This risk of harm, and the impartial claim which it grounds, is one element among others that decides what the vehicle will do.

One final theoretical concern is the Ethical Valence Theory’s ability to predict and react to the occurrence of a second dilemma situation which may result from its original ethically optimal action choice. This relates to the problem of temporal horizons in the vehicle’s decision-making and is as much a technical setback for AVs as it is a foundational theoretical shortcoming in consequentialist ethics. Ideally, the AV should be able to predict and curtail all the negative externalities that could result from its ethical decision-making. Indeed, this may be one of the unspoken assumptions that underpins the idea of autonomous vehicles as super human drivers. Additionally, it may seem that designers have a techno-ethical imperative to implement long temporal horizons into vehicle decision-making, as ‘short-sighted’ AVs may appear comparatively unethical. Nevertheless, the moral importance of a temporal discount rate, and what that rate ostensibly is, are things which become important in the elucidation of appropriate moral profiles, especially if, in a contractualist spirit, these factors are seen to matter to public acceptability. Lacking such information today it is thus not abundantly clear that the functional moral agency of autonomous vehicles is required to extend much past the immediate future, especially if this process threatens the real-time performance of the decision-making algorithm. With these types of concerns, a pertinent boundary is often blurred between the decisional ethics policy of an autonomous vehicle on the one hand, and a larger societal policy for autonomous vehicles, on the other. Two terrains which need not perfectly coincide within the action space of unavoidable accidents. In sum, in dilemma situations, it is essential that the vehicle make the ethically optimal choice within its environment, and ideal that it account for the additional harm it may cause; but it is perhaps only wishful thinking that drives the need for optimization beyond this horizon in the case of autonomous vehicles.

Thus, from a conceptual point of view, the Ethical Valence Theory is designed as an adaptive response to the various types of uncertainty which are characteristic of the early stages of implementation of autonomous vehicles. In a response to moral uncertainty, and a lack of universal ethical consensus, the Ethical Valence Theory provides adjustable and flexible moral profiles in a claim-based structure that is not tightly bound to traditional moral theory, or any single conception of the good. With factual uncertainty within the vehicle’s environment, the EVT proposes valences to help the vehicle deliberate about which facts and features the vehicle must consider in its decisions, information which is bolstered by empirical research. It does not seek to create ‘morally superior robot villains’, nor does it seek to replicate the villainous behaviour of human drivers. Instead, it aims to provide a satisfactory response to the moral and societal concerns of autonomous driving, and an implementable tool for engineers.

## The Computational Implementation of the Ethical Valence Theory

In this section an implementation for the Ethical Valence Theory is discussed. Concepts exposed in the previous sections will be revisited, this time from a computational point of view. Over the course of the many kilometers an autonomous vehicle will drive on public roads, it will occasionally encounter dilemma situations, in which any possible action will result in (potentially lethal) harm to a road user. In our model for autonomous vehicles, the emergence of a dilemma situation triggers an ethically-constrained deliberation model, the Ethical Valence Theory. This model is separate from that which is used in normal conditions, where performance and efficiency constraints guide the decision-making process.

### An Introduction to MDP Algorithms

In this section the composition of a Markov Decision Process (MDP) will be explained. Given the scope of the article, this introduction will be short and simplified, but nonetheless important for the comprehension of later sections. The algorithm is composed of five components (Sigaud and Buffet 2013):

- *State space* ($s_i \in S$) represents all possible AV configurations, thus a sequence of states through time forms its behavior.
- *Action set* ($a_i \in A$) represents the set of all possible actions available for the AV; triggers the transition from one state to another.
- *Transition probability* (*T*) represents the probability whether, given a state, executing an action takes the AV to another state; formulated as $p(s_{t+1}|s_t,a_t)$.
- *Reward function* (*R*) quantifies how good or bad a function is given the defined global objective.
- *Discount constant* ($\gamma$) represents the factor used to adjust the utility at a time $t+1$ to the present (time *t*); defined at the interval [0, 1].

**Fig. 1:**
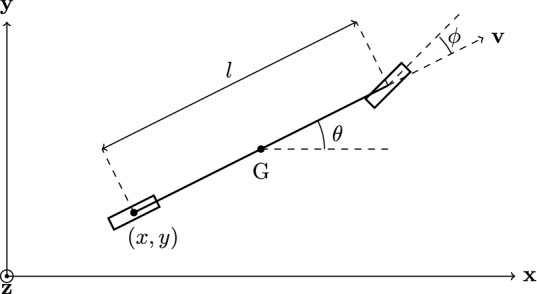
AV’s state representation

For the example that will be used in the application section, the state is defined as $(x,y,\theta ,v,\phi )$, referencing only the AV’s configuration (the configuration of all other road users is already accounted for in the reward function). The couple (*x*, *y*) represents the position of the middle point rear-axis, $\theta$ the direction of the vehicle, *v* the scalar velocity and $\phi$ the steering angle. Figure 1 illustrates all of the mentioned variables using the vehicle.

The output of a MDP algorithm is a policy $\pi ^*$ which, for each state, yields the optimal action to be executed. This action maximizes the value $V(s_t,a_t)$ at the state $s_t$, which is defined by Eq. 1.

1 $$\begin{aligned} V^{*}(s) = \mathbb {E}^\pi \left[ \sum _{i=0}^{\infty }\gamma ^i\cdot r_i(s,a)|s_0=s_i\right] =\max _{a\in A}\left[ r(s,a) + \sum _{s'\in S}p(s'|s,a)V(s')\right] \end{aligned}$$

From Eq. 1 the policy is extracted simply by creating a correspondence between the actions that maximized $V(s_i)$ and $s_i$.

2 $$\begin{aligned} \pi (s) \in \mathbf{argmax} _{a\in A}\left[ r(s,a) + \sum _{s'\in S}p(s'|s,a)V(s')\right] \end{aligned}$$

### Typification of Dilemma Situations

At each step of its trajectory, the AV should be able to tell whether a situation constitutes a dilemma worthy of moral consideration. This situational classification is necessary to determine if the AV must act according to ethical constraints or simply concrete objectives. There are three rules that define the AV’s responsibilities towards the other road users in its environment. If one or more of these rules are violated across all possible actions, this indicates that the non-dilemma portion of the AV’s decision-making cannot cope with the consequences of all possible actions, and thus ethical deliberation is required for the AV to act in an acceptable way. Here harm is defined as the negative consequences suffered by a human after some type of collision with another road user.

**Table:** 

AV duties towards other road users:	
The lives of the passenger(s) must not be put in harm’s way	
The lives of the road users in the environment must not be put in harm’s way	
Traffic regulations must be followed	

Interactions between road users and the AV are covered by the two first rules. For their implementation, vehicles are modeled as rectangles and pedestrians as squares. If, due to the execution of an action, these constructs intercept each other, a collision is considered to have occurred. Eventually, in line with de Moura et al. (2020), a safe frontier around the AV can be defined to discourage the execution of actions which would remove the possibility of breaking without swerving to avoid an accident. However, since there exist other viable actions such a situation would not necessarily constitute a dilemma.

Until this point, adherence to traffic rules and regulations have not been taken into consideration. However, it is certainly desirable that the vehicle should also be sensitive to the interplay between ethical and legal behavior. In this sense, when there is a conflict in dilemma situations between harm to humans on one hand, and adherence to the traffic code on the other, the avoidance of the former should take precedence. As such, the MDP algorithm must be defined so as to express this priority in ways independent from the influence of the temporal discount rate. However, after the transgression, the AV must return to a ‘safe’ state, guaranteeing that another collision does not arise as a direct consequence of its original action choice. If this is the case, then for the AV’s decision-making algorithm all actions, at the first moment, end in collision.

To make the AV conform to traffic laws by design is not a subject that is widely addressed in the literature. Generally, legal conformity presents challenges related to the interpretation of laws which can be vague, admit exceptions, or be internally incoherent; the resolution of all of which may demand some degree of common sense reasoning in order to be solved (Prakken 2017). Additionally, with adherence to traffic laws comes the need to embed relatively abstract norms, used in laws to map concrete behavior, into an AV, and more broadly, into an autonomous system (Leenes and Lucivero 2014). Some authors have already attempted to implement some portions of various traffic codes—related to circulation and behavior—into an autonomous vehicle, such as Rizaldi et al. (2017) (German legislation). Categorically, these attempts have been made using logic-based approaches to emulate constraints, representing only the procedural demands which usually compose a traffic code.

Given these considerations, when the duty obliging traffic code adherence is adequately defined, the entirety of the given traffic code does not need to be exhaustively implemented. Since it is beyond the scope of this article to discuss the methods through which all traffic codes should be implemented within an AV, a set of logical rules will represent the procedural rules present in every traffic code. This set of rules should almost always allow the AV to cruise in a lawful manner. Exceptions to the code and the resolution of conflict between rules will not be covered here, the latter being treated as an ethical decision (even if ideally the procedures to solve conflicts between rules present in traffic codes should be used where possible).

For example, in a straight line domain, without pedestrian strips or semaphores, and with a solid double line the following logic rules can be used:

**Table:** 

Do not cross over into the opposite lane	
Do not drive onto the sidewalk	
Do not surpass the speed limit	

The example above is a simplification which is only valid for a limited number of specific situations. Since the AV should target the 4th or 5th level of automation in generic environments, the actual set of rules will be extended well beyond these three rules.

### The Ethical Deliberation Algorithm

Every action available to the AV is assessed using the defined set of duties. If, at a certain point no acceptable action is available, then the EVT must still choose which action to execute. To do so, it uses two variables: valence and harm. In a general sense, the decisional method proposed here aligns with Bonnemains et al. (2018), since it, too considers world states, decisions (hitherto referred to as ‘actions’) and consequences. However, our approach here differs slightly by proposing a quantification of the consequences of potential actions and, most importantly, accounting for uncertainties in action execution.

#### Defining ‘Harm’

The purpose of considering ’harm’ in ethical deliberation is to measure the risk for AV passengers and other road users involved in a hypothetical collision and thereby ascertaining their claims on the vehicle. Such a measure was proposed in de Moura et al. (2020). For decades, the main variable used to measure collision severity has been the difference of velocity between the two implicated road users ($\Delta v$) (Evans 1994; Jurewicz et al. 2016; Martin and Wu 2018).

Most of the research conducted within the domain of vehicle collisions uses historical accident data to analyze the influence of $\Delta v$ in collisions. To quantify injury, two metrics are popular: risk of fatality and the Abbreviated Injury Scale (AIS) (MacKenzie et al. 1985). The latter will be used here, since it is important to consider not only fatal collisions but those that can inflict severe damage (referred to as MAIS3+, which indicates that at least one injury in some region of the body is above AIS3, a scale going from 0 to 6). In the European Union, this metric is used as a standard to measure road accidents (Weijermars et al. 2018).

All $\Delta v$ used as thresholds for severe injuries are indicated in Table 1, along with their source (typically, an injury is considered as ‘severe’ if it indicates a MAIS3+ injury probability of 10%). For the pedestrian case, the value was obtained from Kröyer (2015), which considers severe injury as having an ISS (Injury Severity Score, defined as the squared sum of AIS for the three most severely injured body regions) larger than 9, which is stricter than MAIS3+. Lateral crashes are covered by near side (driver’s side) and far side (passenger’s side). For single vehicle collisions, the same $\Delta v$ defined for collisions between vehicles is used.

**Table 1:** $\Delta v$ threshold used for fatality collisions

Collision type	Contact	$\Delta v$ value (m/s)	Taken from
Pedestrian collision	–	6.94	Kröyer (2015)
Vehicle collision	Frontal	7.78	Jurewicz et al. (2016)
	Rear	10.56	Jurewicz et al. (2016)
	Near side	5.56	Jurewicz et al. (2016)
	Far side	6.39	Jurewicz et al. (2016)

The data presented in Jurewicz et al. (2016) was collected by the National Highway Traffic Safety Administration (NHTSA), published in Bahouth et al. (2014), considering injuries in the front seat, with a seat-belt, without rollover, with a passenger age ranging from 16 to 55, involving passenger vehicles and heavy vehicles.

This retrospective analysis has some drawbacks. According to Rosen et al. (2011), the data may be biased, since it is only collected across a small set of countries. Also, in the pedestrian case, age is an important feature (Kröyer 2015), therefore its distribution in the studied population plays a role which is unaccounted for in the resulting curve. Underreporting of non-dilemma cases (Martin and Wu 2018), estimation of collision velocities (Rosen et al. 2011), negligence of a vehicle’s mass and geometry (Martin and Wu 2018; Mizuno and Kajzer 1999) and the use of different methodologies to evaluate AIS scores (Weijermars et al. 2018) also reduce the precision of such an approach.

Given that the previous method presents problems when applied to specific situations (despite it generalizing relatively well across a population), accounting for contextual information is necessary. The collision interaction between vehicles can be approximated by a damper-spring-mass system, where the initial velocity of each vehicle is projected onto the axis $\varvec{n}$ (normal to contact plane between both vehicles) and $\varvec{t}$ (tangential to contact plane).

The collision velocity is calculated using the conservation of linear momentum^3^, expressed by Eq. 3. The variable $v_f$, represents the collision velocity for both road users, *k* and *l*. The masses $m_k$ and $m_l$ correspond to the total mass of the road user (if it is a vehicle, then its mass plus the passengers’ mass), and $^{l}{}{\mathbf {v}}_i$ and $^{k}{}{\mathbf {v}}_i$ the velocity and impact of *k* and *l*.

3 $$\begin{aligned} m_{k}^{k}{}{\mathbf {v}}_i + m_{l}^{l}{}{\mathbf {v}}_i = (m_{k}+m_{l})\mathbf {v}_f \end{aligned}$$

In collisions with pedestrians we assume that there is no change in the AV’s velocity, since a vehicle’s mass is much larger than any pedestrian’s. This simplification was adopted considering that the most common variables used to predict injury for pedestrians are the type of vehicle involved (due to the height of bonnet leading edge) (Mizuno and Kajzer 1999; Simms and Wood 2006) along with the vehicle’s impact velocity. The pedestrian’s final velocity is therefore considered equal to the AV’s. For collisions with static objects, the same reasoning which was used with vehicle to vehicle collisions is applied with $v_f$ equal to zero.

Harm, which is the quantification of an accident’s severity, is defined by Eq. 4 (de Moura et al. 2020). For each road user, it is calculated using the velocity variation due to the collision, with velocity at contact for road user *k*, $^{k}{}{\mathbf {v}}_i$ and final velocity $\mathbf {v}_f$. Structural vulnerability is accounted for by $^{k}{}{c}_{vul}$, defined later by Eq. 4. This arrangement accounts for the impact force and the structural vulnerability to such a force.

4 $$\begin{aligned} ^{k}{}{h}(s_t,s_t',a_t) = ^{k}{}{c}_{vul}\cdot \left( \Vert \mathbf {v}_f - ^{k}{}{\mathbf {v}}_i\Vert \right) \end{aligned}$$

Compatibility defines whether two vehicles of different dimensions and masses provide an equal level of security for their occupants (Mizuno and Kajzer 1999). According to Mizuno and Kajzer (1999) and Malczyk et al. (2012), SUVs, for example, protect their passengers but are aggressive towards other vehicles. For pedestrians, the bonnet leading edge height explains why some vehicles are more dangerous for pedestrians than others, since the location of injury depends on which part of the body the vehicle touches (Simms and Wood 2006). The pedestrian may strike the hood in different positions which in turn changes how they are projected onto the ground (Crocetta et al. 2015), causing more or less damage.

All these inherent characteristics are represented by the constant $c_{{\textit{vul}}}$. Ideally, one would calculate $^{k}{}{h}(s_t,s_t',a_t)$ using the same process to determine the probability of MAIS3+ injury versus $\Delta v$ plot (logistic regression with weighting), but velocities at the impact $^{k}{}{\mathbf {v}}_i$ are not available in open databases of vehicle collisions. Additionally, it would be important to classify collisions in terms of the type of vehicle involved (SUV, sedan, mini, etc.) and by the direction of collision (frontal, near side, far side, against a static object, etc.), which are likewise not often available in public databases. As the determination of $^{k}{}{\mathbf {v}}_i$ for collisions itself is the subject of entire projects, it is outside the scope of this paper to discuss it further. As such, the $^{k}{}{h}(s_t,s_t',a_t) = f(c_{{\textit{vul}}},^{k}{}{\Delta v})$ was simplified by a linear function and $c_{{\textit{vul}}}$ will be approximated in the application section.

#### Ethical Valences

The purpose of a valence, as described in previous sections, is to represent the degree of social acceptability that is attached to the claims of the road users in the vehicle’s environment. In this sense, the claims of certain road users can be more or less ‘acceptable’ to satisfy via the vehicle’s action selection. The valences, in so far as they are rooted in the phenomenal signature of individuals, then track various physical characteristics which are seen to carry social importance: height, age, gender, helmet-wearing-cyclist, or stroller-pushing-adult, all of which are detectable by the object classification algorithms of the AV. Importantly, the determination of the strength of these valences is accomplished through a type of ranking or hierarchisation, which associates a road user’s claim with a certain class or category of valence, as shown by Table 2. In this way, depending on the amount or detail of the valence features under consideration, there can be more or less valence categories.

**Table 2:** Possible valence hierarchy

Feature 1	Feature 2	Classification
Young (0–18 years)	Pedestrian	A
Old (65+ years)	Pedestrian	B
Young	Vehicle passenger	C
Old	Vehicle passenger	D
Adult (18–65 years)	Pedestrian	E
	Vehicle passenger	F

In this example for instance, two features are used: age and type of road user. The classification was created considering recent studies which suggest that western societies prefer to spare the young and vulnerable (understood in terms of exposure to injury) in AV collisions (Awad et al. 2018). In the case of multiple people, vehicles or agglomeration of pedestrians, the entity that has the larger number of users with a high classification has the preference. Between an AV with passengers C and F and another with C and D, the latter is considered to have a higher valence.

Importantly, in cases where the chosen valence features are minimal or simple (such as in the example above) the likelihood that multiple road users will have the same valence, but differing claims, increases. In this sense, there may be certain situations wherein the harm measurement becomes the decisive factor in action selection. In these cases, the vehicle satisfies the strongest claim in its environment, protecting the person whose welfare is most severely impacted, due either to a dangerous context (high velocity difference) or to an inherent vulnerability (detected by the structural vulnerability constant). This simple maximization of welfare, however, is complicated by the operational moral profile, which specifies the claim mitigation process between those passengers inside the car, and those road users outside of it. To this end, two possible moral profiles can be seen in Table 3. Risk is considered severe if $\Delta v$ surpasses the limits defined in Table 1.

**Table 3:** Possible moral profiles for an AV

Moral profiles	Definition
Risk averse altruism	Protects the road user with the highest valence as long as AV passenger’s risk is not severe
Threshold egoism	Protects AV passengers as long as risk for other road users with higher valence than the AV is not severe

None of these profiles perfectly resemble any traditional moral theory, or if anything, resemble various positions along the spectrum of egoistic rationality (Parfit 1984). This is intentional, as these profiles are designed to capture various degrees of compromise between the claims and valences of the AV’s passengers and those of the other agents within the AV’s environment. These profiles often reinforce the idea that a certain degree of morally admirable partiality is possible, or perhaps even necessary in AV behavior, in order to best align with user expectations, or to garner user trust (Gerdes and Thornton 2015; Keeling et al. 2019). The profiles listed in Table 3 are likewise non-exhaustive and represent somewhat factually opaque renditions of the profile types the Ethical Valence Theory can accommodate. In these versions, the role of the harm calculation is important, as it is the principal factor which informs the various consequences of the AV’s actions, due to trade-offs between the passenger(s) claims and those of the other agents in the vehicle’s environment.

#### Ethical Deliberation

Once informed by the valences and harms, the AV can deliberate on an action, a step which is crucially guided by the operational moral profile. Each moral profile indicates a unique form of deliberation, as shown in Table 4. It is perhaps worth restating that the moral profiles—and for that matter, ethical deliberation itself—is only present in the vehicle’s tactical planning in dilemma situations. Otherwise, concrete, goal-driven planning is operative, using standard decision-making criteria.

**Table 4:** Optimization procedure based on the moral profile chosen

Moral profiles	Deliberation
Risk averse altruism	Minimize the expected harm of the road user with the highest valence until the AV’s collision becomes severe
Threshold egoism	Minimize the expected harm of the AV until the risk to a road user with a higher valence becomes severe

Each profile requires a different implementation. Using the risk-averse altruism case as an example, to deliberate, the AV’s state ($s_i$, represented by $(x_i,y_i)$, position, $\theta _i$ direction, $v_i$ velocity and $\phi _i$ steering angle), environment state (*e*, which contains the position and velocity of all agents in the environment), highest road user valence ($\eta$) and maximum $\Delta v$, are the input. The action that should be executed ($a_{\eta }$), is the output. As a first step, all harm measurements for possible actions and the proceeding states (represented by the state space $S'$, composed by the states reached after one single transition) need to be calculated. Here the decisional horizon is equal to one transition, since the accident will follow immediately afterwards.

This is done first by solving Eqs. 3 and 4. Only one road user is implicated with the AV in an ideal collision. All the other road users are taken into account using the transition uncertainties, represented by $p(s_i'|s_i,a_j)$, given the actual state ($s_i$) and an action ($a_j$). 
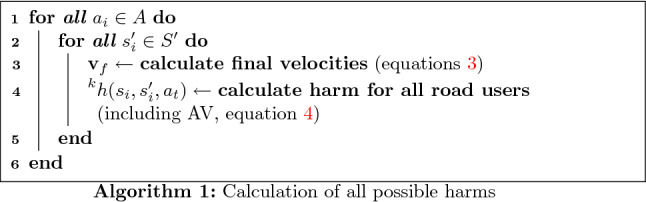


If all possible outcomes produce a velocity difference which is larger than $\Delta v$ (the road user’s velocity minus the AV’s predicted velocity), then the collision is severe and the safety of the AV’s passenger is prioritized. In the considered profile, the chosen action minimizes the expected harm for the AV. It should be pointed out that $\Delta v$ changes according to collision type (as can be seen in Table 1). The transition probability is used to calculate the expected harm ($h_{\mathrm{exp}}(s_i,a_j)$, Eq. 5), which represents a mean harm value for a road user k, given that for one state $s_i$ and action $a_j$ different states $s_i'$ can be reached, and therefore different collisions can happen. The position of all road users and the observation of the AV’s state is considered to be perfect (no uncertainty in these measures).

5 $$\begin{aligned} ^{k}{}{h_{\mathrm{exp}}}(s_i,a_j) = \sum _{s_i'\in S'}p(s_i'|s_i,a_j)h(s_i,s_i',a_t) \end{aligned}$$

**Fig. 2:**
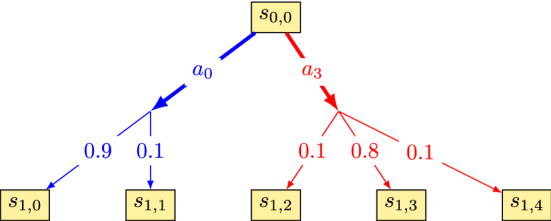
State transition uncertainty for $a_0$ and $a_3$

The transition probability can represent the estimation uncertainty about the behavior of the other road users, among other sources of uncertainties. Since the MDP algorithm described here is not concerned with such estimations, the transition probability will be static values, depending on the action and the current state. Each action will have a probability of 0.8 to succeed and 0.2 to take the AV to the neighbor states (0.1 for each). For example, in Fig. 2, action $a_3$ has 0.8 of chance to take the AV from $s_{0,0}$ to $s_{1,3}$, and 0.1 of chance to take it either to $s_{1,2}$ or $s_{1,4}$. For the extremity actions, the probability becomes 0.9 to succeed and 0.1 to the neighbor state (case of action $a_{0}$ in Fig. 2).

If the set of admissible actions according to $\Delta v$, $A_{\eta }$, is not empty, the chosen action minimizes the road user’s expected harm with the highest valence for the actions $\in A_{\eta }$. If multiple minimal actions exist, then the one that minimizes the AV’s expected harm is chosen. This process is shown by algorithm 2. 
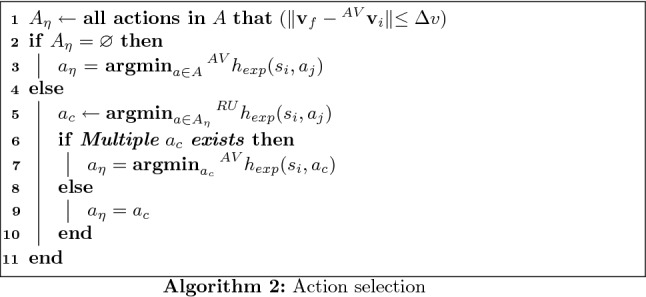


Passing from the AV’s harm minimization to the road user’s harm minimization may appear to be an extreme position in comparison with other alternatives, such as the possible minimization of both quantities. An infinite number of compromises can be imagined between the AV and road users, however in our examples here both moral profiles oppose each other to maximize the safety of only one road user. For the threshold egoism profile, only the action deliberation process shown by algorithm 2 would change.

### Application of EVT in a Hypothetical Situation

**Fig. 3:**
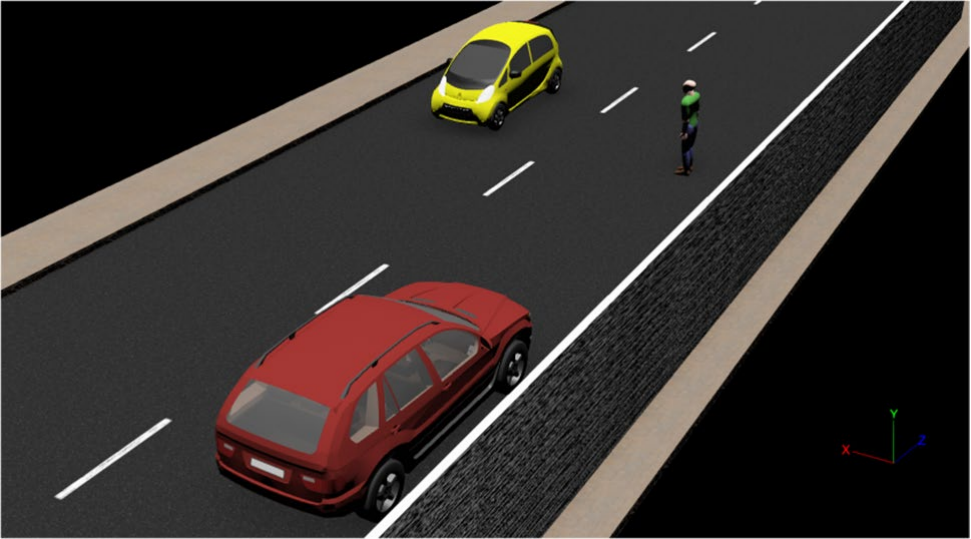
Possible dilemma situation

In Fig. 3 a simplified dilemma situation in an urban environment is presented. From the action set, only three actions stand out: swerve to the left and hit the CitrÖen CZero, go straight and hit the pedestrian, or swerve to the right and hit the wall. The action space is searched to find the best actions, and in this case only three actions have different consequences. Therefore, the EVT must be mobilized to guide the decision process.

**Fig. 4:**
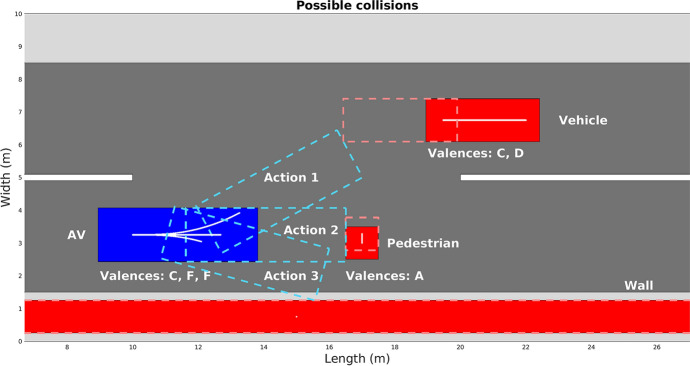
Collision simulation for situation 1

Figure 4 shows the collision simulation when the AV’s initial state is (10, 3.25, 0, 15, 0), (*x*, *y* coordinates of the vehicle, direction, longitudinal velocity and steering angle), hereby defined as *situation 1*. To simulate the AV behavior, the non-holonomic single track model (Qian et al. 2016) was used; the collision happens inside a decision iteration, which divides the AV’s trajectory in periods of 0.5 seconds.

To calculate the vulnerability constant, $c_{{\textit{vul}}}$, the data available in Kröyer (2015) and Jurewicz et al. (2016) are used with Eq. 6, $\hbox {Prob}_{{\textit{MAIS}}3+}(\Delta v)$ being the probability of MAIS3+ injury given a $\Delta v$, difference of initial velocities before the collision. Admittedly, this is an imperfect way to account for such parameters (as discussed in previous sections), but for the example presented it will suffice.

6 $$\begin{aligned} c_{vul} = \frac{1}{1-\text {Prob}_{MAIS3+}(\Delta v)} \end{aligned}$$

Table 5 shows the preference order, given the valences for each road user in Fig. 4.

**Table 5:** Valence hierarchy

Road user	Valences	Classification
AV	C, F, F	$3^{\circ }$
Vehicle	C, D	$2^{\circ }$
Pedestrian	A	$1^{\circ }$

In situation 1, $\Delta v$ are equal to 23.1 m/s for AV-vehicle (frontal collision), 14.1 m/s for AV-pedestrian (pedestrian collision) and 14.2 m/s for AV-wall (frontal collision). Comparing these values with the limits established in Table 1, one can conclude that all actions pose a serious risk for the AV and all other road users. Following the risk-averse altruism profile would entail choosing to run over the pedestrian, since the AV must be prioritized ($\Delta v$ is above the limit, therefore the AV’s harm is minimized, selecting the bold value in Table 6; such a procedure is seen in algorithm 2). Table 6 shows the harm and expected harm (sum of harms weighted by transition probability, Eq. 5) calculated for the AV in each possible collision.

If the AV is configured to have threshold egoism as its operational moral profile, the choice would be to collide with the wall, since the pedestrian’s and vehicle’s valences are higher, according to the Table 5 (both $\Delta v$ are above the limit, thus the road users with valences higher than the AV have their expected harm minimized, resulting in the italic values at Table 7). Table 7 presents in its first column the nominal road user’s harm, while in the second and third columns the vehicle’s expected harm and the pedestrian’s expected harm, obtained using the transition probability by Eq. 5. Since the wall is a static object, its harm and expected harm is zero (only human safety is considered; historical, cultural or affective value to a static object like a tree or a monument are disregarded).

**Table 6:** AV’s harm for each possible collision for situation 1

	AV’s harm	AV’s exp. harm
Veh. col.	8.77	7.02
Ped. col.	0	**2**.**46**
Wall col.	15.80	12.64

**Table 7:** Road users’s harm for each possible collision for situation 1

	Road user’s *h*	Vehicle’s $h_{\mathrm{exp}}$	Pedestrian’s $h_{\mathrm{exp}}$
Veh. col.	16.80	15.12	1.57
Ped. col.	15.71	1.68	12.57
Wall col.	0	*0*	*1*.*57*

**Fig. 5:**
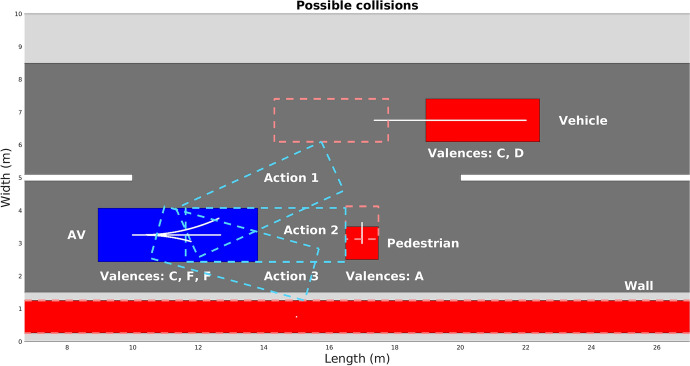
Collision simulation for situation 2

Figure 5 shows *situation 2*, where the initial AV’s state is (10, 3.25, 0, 7.5, 0) (and and the respective positions of the road users are shown in Table 11 at Appendix 6.1), harms and differences in velocities would invert the chosen action. Velocity differences would be 14.87 m/s, 5.63 m/s and 6.07 m/s respectively, meaning that a collision with the pedestrian and with the wall do not surpass the severe threshold.

Using risk-averse altruism as the operative moral profile results in the wall collision action being executed (the road user that has the highest valence has its expected harm minimized), resulting in the action represented by the italic value in Table 8 and for the threshold egoism the chosen action would be collision with the pedestrian (In this case the AV’s expected harm would be minimized), resulting in the bold value at Table 9. Tables 8 and 9 are analogous to Tables 6 and 7, respectively.

**Table 8:** Collision quantification for situation 2

	AV’s harm	AV’s exp. harm
Veh. col.	5.10	4.08
Ped. col.	0	*1*.*12*
Wall col.	6.07	4.86

**Table 9:** Quantification for other road users in situation 2

	Road user’s *h*	Vehicle’s $h_{\mathrm{exp}}$	Pedestrian’s $h_{\mathrm{exp}}$
Veh. col.	10.85	9.76	0.56
Ped. col.	5.63	1.08	4.51
Wall col.	0	0	**0**.**56**

## Conclusion

The research explored in this article, and the moral and computational approach that it underpins, should not be seen as an ‘ultimate’ normative answer to behavior in autonomous vehicles. To this end, there are a number of reasons why the Ethical Valence Theory might fail to meet the expectations of certain stakeholders in the development of autonomous vehicles. Firstly, the Ethical Valence Theory quite clearly discriminates between different road users, for instance by identifying the ‘passenger’ as distinct from other vulnerable road users, or by distinguishing the type of vehicle(s) that will be involved in a dilemma situation. This positioning may be seen as problematic for some, since it fails to adhere to some of the prominent normative doctrines that have been proposed in recent years (Luetge 2017), most of which condemn the practice of discrimination between potential victims of an AV’s actions. This concern might in turn be compounded by the ambiguity of ‘valences’. How can we ensure that the data which is used to inform them is fair and representative, and what to do if the data collected threatens to undermine civic or human rights? The construction of the deliberation process of highly autonomous systems such as autonomous vehicles will likely remain a polemic subject in the years to come. It will require both a high degree of interdisciplinary cooperation between scientific fields which have enjoyed longstanding autonomy, as well as a steep learning curve on the part of the users, states and institutions of the societies in which these technologies will be implemented.

What is clear at this juncture, is that when technologies make autonomous decisions that have an impact on the lives and welfare of human beings, designers have a corresponding responsibility to ensure that that the decisions made are acceptable, ethical and respectful, rather than simply efficient. Part of this challenge can be answered through law, and more still through ethical considerations and moral theory, however the final decisions must ultimately be representative of the people they effect, their values, claims and conceptions of the good. The main goal of the Ethical Valence Theory is to attempt to embrace this multidisciplinary and urgent need for public involvement and approval, by providing the groundwork for the design of an ethical and acceptable autonomous vehicle for the world’s roads.

## Appendix

### Constants Used in Simulation

See the Tables 10 and 11.

**Table 10:** Data about road users

Model	m (kg)	Dimensions $(\hbox {m} \times \hbox {m})$
AV–BMW X5	2105	$4.853 \times 1.65$
Citroen CZero	1065	$3.475 \times 1.310$
Pedestrian	80	$0.625 \times 0.625$

**Table 11:** Road users’ initial state

Road user		Situation 1	Situation 2
Vehicle	($x,y,\theta ,v,\phi$)	(25, 6.75, 180, 15, 0)	(22, 6.75, 180, 7.5, 0)
Pedestrian	($x,y,\theta ,v$)	(17, 3, 90, 1.5)	(17, 3, 90, 1.5)
Wall	($x,y,\theta ,v$)	(15, 0.75; 0, 0)	(15, 0.75, 0, 0)

## Footnotes

1 *Important notice*: This work is to be understood as a “what if” study exploring how AVs would make their decisions if the EVT method were adopted and what the consequences might be. The particular settings (or “moral profiles”) that we propose in the course of the article to illustrate the functioning of the method are not prescribed as the only possible solutions (if any) to the ethical problems that can arise in automated driving in real situations. Our aim is rather to propose a general approach that could help legislators or regulators in their assessment about automated driving deployment.

2 The term ‘valence’ has precedent in moral philosophy. Traditionally, the valence of a reason determines whether it counts in favour of or against an act or judgement-sensitive attitude, and indicates its positive or negative contribution to the overall moral status of the act (Copp 2005). Our use of the term ‘valence’ departs from this standard account, and rather denotes the degree to which the identity of a specific claimant satisfies a number of acceptability-focused criteria. In this sense, the more ‘acceptable’ a particular individual’s claim is (the more she satisfies whichever acceptability criteria are being used), the stronger the valence. Metaphorically, if a claim represents the voicing of the preference of a given individual in the vehicle’s deliberation about which action to pursue, the valence determines how loudly the vehicle hears it.

3 The mechanics of a collision are more complex, involving both road user’s geometry and dissipation forces, from sound and temperature to plastic deformation. The same model from de Moura et al. (2020) is used to calculate the final velocity, approximating the road users as a punctual mass.
